# How to prevent cognitive overload in the walking-arithmetic dual task among patients with Parkinson’s disease

**DOI:** 10.1186/s12883-023-03231-5

**Published:** 2023-05-25

**Authors:** Ying Xu, Canru Geng, Tong Tang, Juanying Huang, Ying Hou

**Affiliations:** 1 Department of Rehabilitation Medicine, The Affiliated Suzhou Hospital of Nanjing Medical University, Suzhou Municipal Hospital, Gusu School, Nanjing Medical University, No. 286 Guangji Road, Suzhou, Jiangsu 215000 China; 2 Department of Neurology, The Second Affiliated Hospital of Soochow University, No. 1055 Sanxiang Road, Suzhou, Jiangsu 215000 China

**Keywords:** Parkinson’s disease, Cognition, Executive function, Gait

## Abstract

**Background:**

Participants with Parkinson’s disease (PD) may experience difficulty during certain dual-task (DT) tests. Thus, it is necessary to keep the cognitive load within the limits of their ability.

**Objective:**

To identify cognitive overload and its influence on the walking and auditory addition and subtraction (AAS, all values within the range of 0–20) DT performance of patients with PD.

**Study design:**

A cross-sectional observational study with convenience sampling.

**Setting:**

Outpatient clinic of the Department of Neurology.

**Subjects:**

Sixteen patients with PD and 15 sex- and age- matched people elderly healthy controls (HCs).

**Methods:**

Verbal calculation responses and gait parameters were collected from the two groups in the 2-min single arithmetic task (2-min SAT), 2-min single walking task (2-min SWT), and 2-min walking–arithmetic dual task (2-min WADT).

**Results:**

The group differences in the lower-limb gait parameters increased in the 2-min WADT (*P* < 0.01), and those in the arm, trunk, and waist parameters did not change (*P* > 0.05). In the 2-min SAT, the calculation speed of the PD group was significantly lower than that of the HC group (*P* < 0.01). In the 2-min WADT, both groups made more errors (*P* < 0.05), especially the PD group (*P* = 0.00). PD group miscalculations occurred in the first half of the 2-min SAT but were uniformly distributed in the 2-min WADT. The HC group and PD group had subtraction self-correction rates of 31.25% and 10.25%, respectively. The PD group tended to make subtraction errors when the value of the first operand was 20 or 13.46 ± 2.60 and when the value of the second and third operands were 7.75 ± 2.51 (*P* = 0.3657) and 8.50 ± 4.04 (*P* = 0.170), respectively.

**Conclusions:**

Cognitive overload was observed in patients with PD. This was mainly reflected in the failure of gait control and accurate calculation, indicated by gait parameters of the lower limbs and accuracy of calculation. To impose a constant cognitive load, the amount added or subtracted, especially in subtraction with borrowing, should not be mixed during a sequential arithmetic problem in the DT, and equations with the value of the first operand equal to 20 or approximately 13, the value of the second operand approximately 7, or the value of the third operand of approximately 9 should be excluded in the AAS DT.

**Trial registration:**

Clinical trial registration number: ChiCTR1800020158.

## Introduction

Parkinson’s disease (PD) is a progressive neurodegenerative disease with motor and nonmotor symptoms [1, 2]. Patients with PD exhibit both bradykinesia and cognitive impairment, which seriously reduce their quality of life [3]. Previous studies have demonstrated that gait disorder in PD does not independently result from musculoskeletal impairment but is also related to cognitive impairment [4, 5]. Therefore, the dual-task (DT) paradigm [6] is the best tool to assess the bidirectional influence of cognitive and motor function in patients with PD. The gait and cognitive performance of patients with PD in the DT differ significantly from those of healthy controls (HCs) [7, 8]. At present, the DT is widely used as a combination of an experimental approach and treatment of executive function in patients with PD [9, 10].

The arithmetic task is one of the most commonly used cognitive components of the DT [11]. Its cognitive load varies according to the numerical magnitude of each operation. The numerical magnitude of most widely used formats, such as serial seven-subtraction or three-subtraction, is not easy to keep consistent. This deficiency is aggravated by miscalculations in the series [12]. As a result, the cognitive load of the motor–arithmetic DT during assessment or treatment might fluctuate and overload participants, especially those with impaired executive function. This cognitive overload may result in failure to complete the DT, which is not easy to identify or prevent. Therefore, awareness of the difficulty of different components of motor–arithmetic DTs are necessary for maintaining a cognitive load within the limits of participants’ cognitive ability.

However, the relative difficulty level of arithmetic problems with different numbers remains unclear in patients with PD. Our aim was to determine the relative difficulty level of arithmetic problems for patients with PD by observing their DT performance, such as miscalculations, time delays, and gait changes [13]. Thus, we may prevent the cognitive overload of patients with PD by choosing suitable arithmetic problems for the motor–arithmetic DT.

In this study, we used the motor–arithmetic paradigm, involving walking with random auditory addition and subtraction (AAS; all values within the range of 0–20) as the DT, based on auditory addition task design [14, 15]. The performance of patients was observed in real time. The results provide preliminary evidence of how to prevent cognitive overload during the walking–arithmetic DT among patients with PD.

## Methods

This was a preliminary cross-sectional observational study with convenience sampling and an age- and sex-matched control group. Motor and cognitive performances were observed to confirm cognitive overload manifestations and their influencing factors. The trial was registered with the Chinese Clinical Trial Registry [No. ChiCTR1800020158(18/12/2018)].

### Participants

Patients in the PD group were recruited from the outpatient clinic of the Department of Neurology of the Second Affiliated Hospital of Soochow University. The inclusion criteria were as follows: 1) a clinical diagnosis of idiopathic PD; 2) PD stage 1–3 on the Hoehn and Yahr (H & Y) scale; 3) no motor response fluctuations; 4) a Mini-Mental State Examination (MMSE) [16] score of ≥ 24; 5) ability to walk independently for more than 2 min; and 6) an age of 60 − 75 years. The exclusion criteria were as follows: 1) a history of brain surgery; 2) clinically significant comorbidities likely to affect gait and cognition (including diabetes mellitus, rheumatic disease, musculoskeletal disease, cardiovascular disease, respiratory disease, cerebrovascular disease, or other neurological diseases); 3) major depression; or 4) uncorrected vision or hearing problems.

All participants had previously been diagnosed according to the Movement Disorder Society (MDS) clinical diagnostic criteria [17] upon admission by a specialized medical doctor in the Department of Neurology. All participants and their legal guardians gave the informed consent before test. A total of 16 patients with PD were enrolled in the study, and 15 sex- and age-matched healthy individuals were also enrolled (HC group).

The study was explained to patients and their caregiver(s), and informed written consent was obtained from all participants before participation in this study. The research protocol was approved by the Human Studies Committee of the hospital and was carried out according to the ethical standards of the 1964 Declaration of Helsinki and its subsequent amendments.

### Experimental protocol

**Fig. 1:**
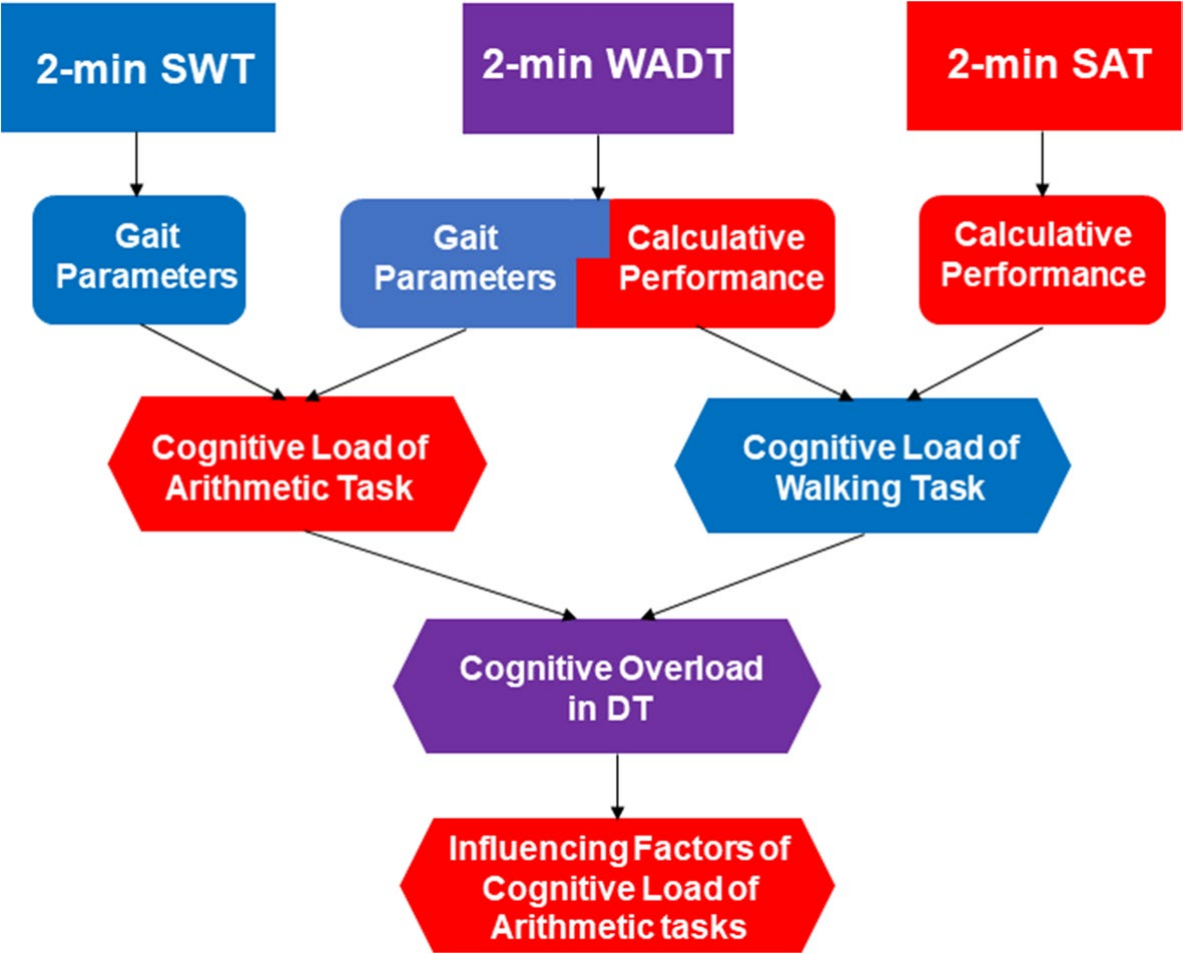
Flowchart of the experimental procedures and data collected

Every participant completed the 2-min single arithmetic task (2-min SAT), 2-min single walking task (2-min SWT), and 2-min walking–arithmetic dual task (2-min WADT). The patients started the tests during the ‘‘ON’’ state of the medication cycle, approximately 45 min to 1 h after the last intake of antiparkinsonian medication; patients confirmed that they were in a good ‘‘ON’’ state according to their own subjective scale. The clinical evaluation was conducted first and was followed by the 2-min SAT, 2-min SWT, and 2-min WADT, completed in a randomized order. A 5-min break was provided between each assessment. The entire process lasted approximately 20 min. Figure 1 presents an outline of the experimental procedures.

### Clinical assessments

Data was collected in terms of participant age, sex, MMSE score, Montreal Cognitive Assessment (MoCA) [16] score, Pittsburgh Sleep Quality Index (PSQI) [18], Hamilton Rating Scale for Depression (HRSD) score, and Hamilton Anxiety Scale (HAMA) [19] score. In addition, the Unified Parkinson’s Disease Rating Scale (UPDRS) [20], H&Y staging [9], and Freezing of Gait Questionnaire (FOGQ) [21] were used to quantify the severity and extrapyramidal signs of PD. Assessments were performed by a neurology physician (permanent staff).

### The 2-min SAT

A list of arithmetic problems was prepared for the participants before the tests. The numbers (operands) and operation (addition or subtraction) were randomly generated in Microsoft Excel, and each of the three operands in an equation was ≤ 20. According to the operation and operands, five types of mental calculations were assessed: single-digit addition, single-digit subtraction, addition with carrying, subtraction with borrowing, and ties (two additions equal in magnitude). Participants were asked to perform the 2-min SAT while comfortably seated in a silent room. This task involved listening to a series of randomized verbal addition and subtraction problems, with all numbers in the range of 0–20, from a female experimenter, and to give their answers verbally and as rapidly as possible over 2 min. The process was recorded with an audio recorder. The accuracy and speed of the calculation, number of errors, delay in calculation, repetitions of the question, and so on, were recorded.

### The 2-min SWT

The 2-min SWT was assessed in an obstacle-free, 25-m long, 4-m wide corridor. The participants walked for 2 min at a comfortable pace without performing any other tasks and were allowed to turn at the end of the corridor. Gait parameters were measured by wearable inertial sensors.

Gait parameters were recorded by the Ambulatory Parkinson Disease Monitoring (APDM) system (Mobility Lab v2, APDM, Portland, USA) [22]. The system consists of a computer, Bluetooth receiver, and six inertial sensors (5 cm × 5 cm × 1.5 cm; 50 g) carried on the wrists, metatarsals (i.e., on the middle of the top of the foot), chest, and waist. Data are delivered from the sensors to the computer by a Bluetooth receiver in real time. During the 2-min walking measurement, over 20 parameters were recorded from the two sides in each gait cycle, including cadence, double support, gait cycle duration, gait speed, elevation in midswing, lateral step variability, circumduction, foot strike angle, toe-off angle, single limb support percentage, stance, step duration, stride length, swing percentage, terminal double support percentage, toe-out angle, range of motion of lumbar and trunk (coronal, sagittal, and transverse), arm swing velocity and range of motion, angle, velocity, steps in turn, initial contact, toe-off, and midswing. The measurements were developed by an engineer and neurology physician, and abnormal performance, such as stopping walking and or forgetting the tasks, was recorded.

### The 2-min WADT

During the 2-min WADT, participants were asked to listen to and verbally answer arithmetic problems while walking. Answers to the AAS problems were recorded with an audio recorder, and the gait parameters were assessed using APDM. If the participants unexpectedly stopped walking or answering the problems, the experimenter would remind them once after 3 s. If an verbal reminder was not effective, the patient was withdrawn from the study.

### Statistical analysis

Statistical analyses were performed using SPSS v.24 (SPSS, Chicago, IL, United States). We first explored dependent variables to determine missing data points, normality of distributions (with Kolmogorov–Smirnov tests), and outliers (using the Explore command of SPSS v.24). An alpha level of 0.05 was used for all statistical tests. Comparisons of continuous variables between the two groups were conducted using analyses of variance (ANOVAs), and categorical variables were compared using the chi-square test.

## Results

The clinical assessments of the participants are shown in Table 1. Calculation results and gait parameters were collected without any missing data. The two groups completed all tasks safely and successfully during the experiment. Furthermore, patients’ verbal repetition of auditory tasks was observed in both groups.

**Table 1:** Subject characteristics of the two groups

	All, *N* = 31	HC group, *n* = 15	PD group, *n* = 16	*P* (HC group vs. PD group)
Age (y)	63.55 ± 8.31	61.47 ± 5.56	65.50 ± 10.04	0.17
Sex (M/F)	17/14	8/7	9/7	0.87
Disease duration (y)	-	-	6.19 ± 3.85	-
MMSE score	27.61 ± 3.48	29.67 ± 0.72	25.69 ± 3.94	0.00
MoCA score	24.90 ± 4.25	28.00 ± 2.03	22.00 ± 3.70	0.00
H & Y stage (II/III)	-	-	1.85 ± 0.59	-
UPDRS score	-	-	32.75 ± 12.37	-
UPDRS-I score	-	-	2.75 ± 2.04	-
UPDRS-II score	-	-	10.56 ± 5.89	-
UPDRS-III score	-	-	18.00 ± 5.85	-
UPDRS-IV score	-	-	1.44 ± 2.06	-
FOGQ score	-	-	6.75 ± 5.04	-
HAMA score	-	-	10.25 ± 8.59	-
HRSD score	-	-	10.19 ± 6.86	-
PSQI score	-	-	9.19 ± 4.69	-

*Abbreviations: MMSE* Mini-Mental State Examination, *MoCA* Montreal Cognitive Assessment, *H & Y scale* Hoehn and Yahr scale, *UPDRS* Unified Parkinson’s Disease Rating Scale, *FOGQ* Freezing of Gait Questionnaire, *HAMA* Hamilton Anxiety Scale, *HRSD* Hamilton Rating Scale for Depression, *PSQI* Pittsburgh Sleep Quality Index

### Gait parameters of the two groups in the 2-min SWT and 2-min WADT

**Fig. 2:**
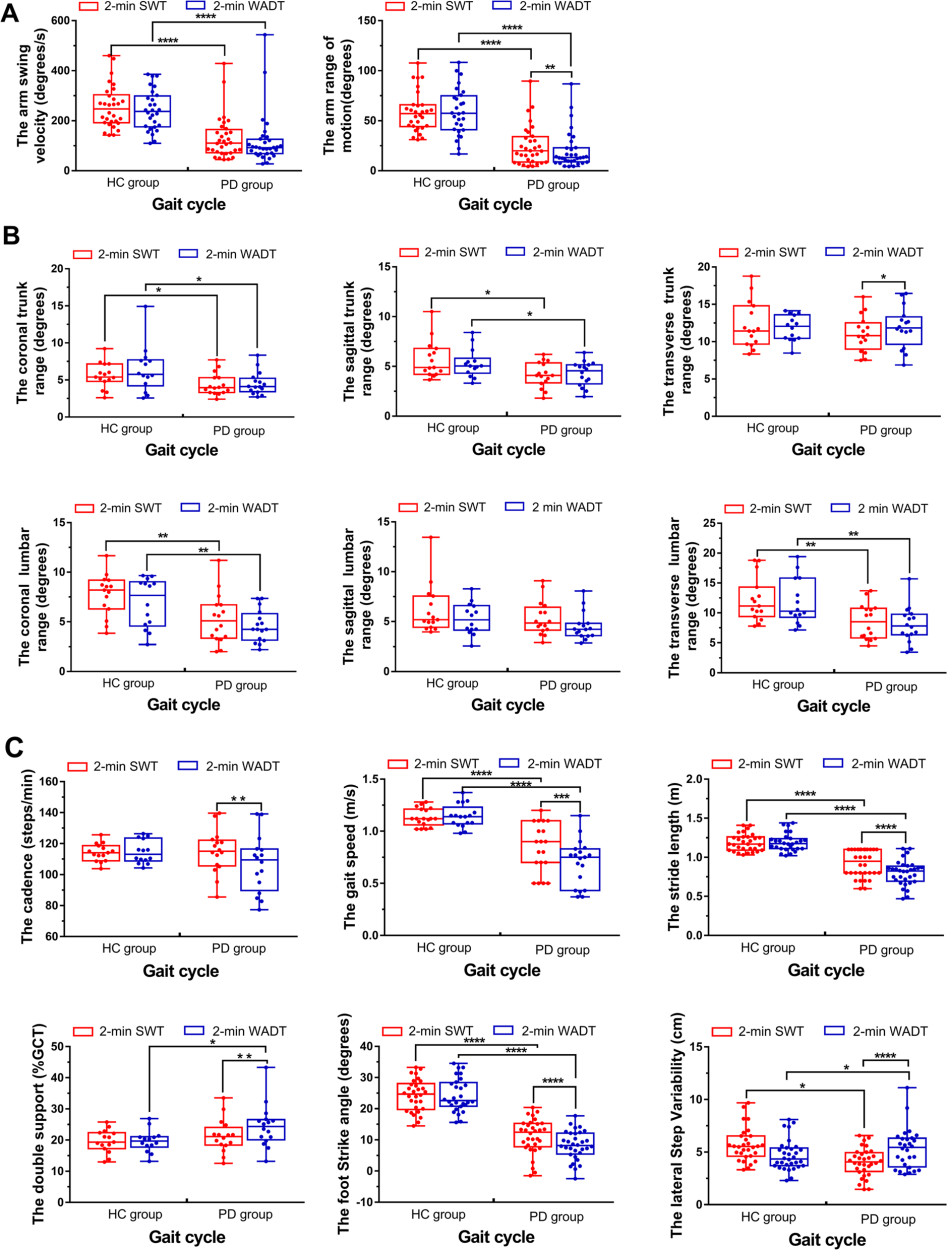
Gait parameters of the two groups in the 2-min SWT and 2-min WADT. *, *P* < 0.05; **, *P* < 0.01; ***, *P* < 0.001; ****, *P* < 0.0001

The two groups completed the walking task without interruption in the 2-min SWT and 2-min WADT, except for two patients with PD who stopped walking during the 2-min WADT and continued after verbal reminders. Most of the gait parameters of the PD group were significantly worse than those of the HC group (*P* < 0.01). The group differences in the gait parameters of the upper limbs, trunk and waist, and lower limbs are shown in Fig. 2.

### Speed and accuracy of calculation of the two groups in the 2-min SAT and 2-min WADT

In the 2-min SAT, the two groups exhibited accurate calculation, but the calculation speed of the PD group was significantly less than that of the HC group (*P* < 0.01). In the 2-min WADT, the amount of problems completed (hereafter, amount) and calculation speed were not significantly reduced (*P* > 0.05). However, the accuracy rates of the two groups decreased significantly (*P* < 0.05), especially that of the PD group (*P* = 0.00). The differences between the two groups in terms of amount, speed, and accuracy were significant (see Table 2).

**Table 2:** General arithmetic results of the two groups

	HC group, *n* = 15			PD group, *n* = 16			*P* (HC group vs. PD group; 2-min SAT)	*P* (HC group vs. PD group; 2-min WADT)
	2-min SAT	2-min WADT	*P* (2-min SAT vs. 2-min WADT)	2-min SAT	2-min WADT	*P* (2-min SAT vs. 2-min WADT)		
Amount of problems (n)	57.67 ± 6.42	53.40 ± 8.85	0.14	35.19 ± 8.60	29.25 ± 10.83	0.09	0.00**	0.00**
Calculation speed (n/min)	28.83 ± 3.21	26.70 ± 4.42	0.14	17.60 ± 4.30	14.63 ± 5.41	0.09	0.00**	0.00**
Calculation accuracy (%)	1.00 ± 0.00	0.99 ± 0.01	0.03*	0.99 ± 0.02	0.92 ± 0.06	0.00**	0.05	0.00**

^*^, 0.01 < *P* < 0.05; ^**^, *P* < 0.01

### Percentages of incorrectly answered problems

**Fig. 3:**
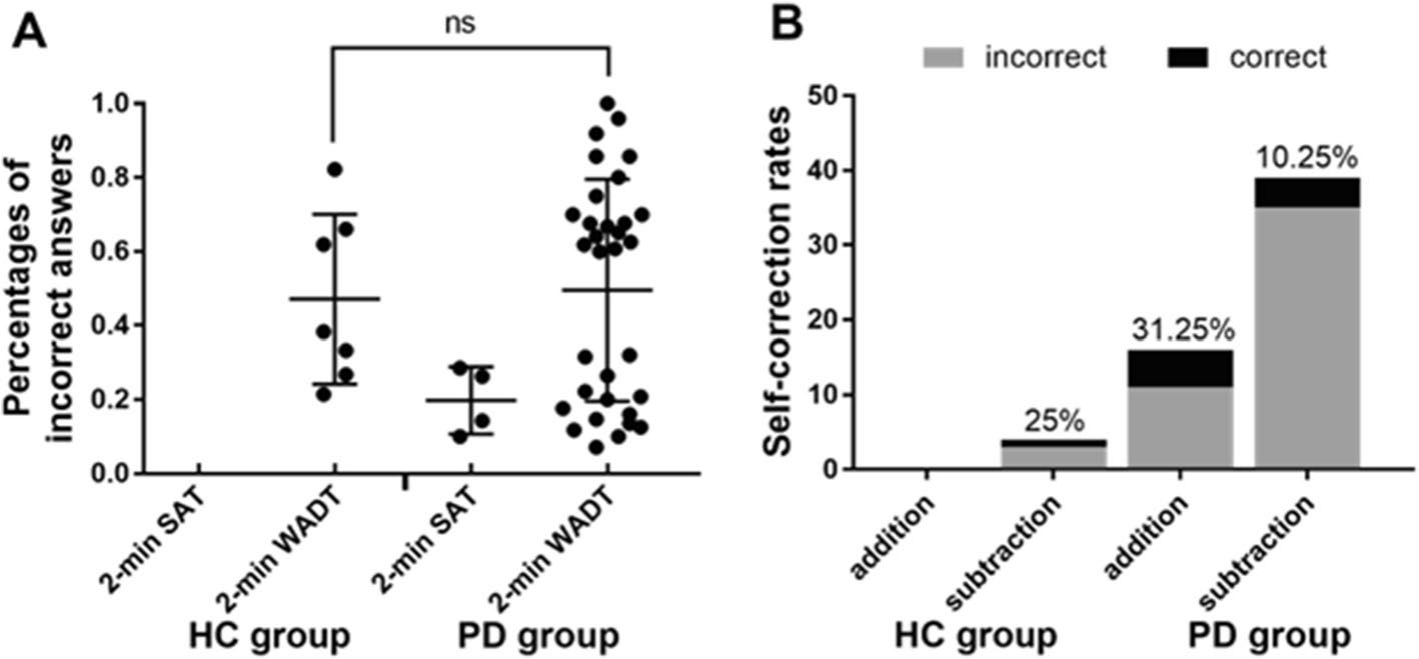
Characteristics of the incorrect answers of the two groups. **A** Percentages of incorrect answers. **B** Self-correction rates. ns: *P* > 0.05

In the 2- min SAT, the percentage of incorrect answers in the PD group was 19.79 ± 7.84%. In the 2-min WADT, the percentages of incorrect answers in the HC group and the PD group were 47.15 ± 21.21% and 49.57 ± 29.52%, respectively; incorrect answers were nonnormally distributed, and the group difference was not statistically significant (*P* = 0.95) (see Fig. 3A).

### Arithmetic types of incorrectly answered problems

The two groups exhibited more errors in subtraction. The HC group exhibited only errors in subtraction, with a self-correction rate of 25%. The PD group exhibited errors in both addition and subtraction but exhibited more errors in subtraction, with self-correction rates of 31.25% and 10.25%, respectively (see Fig. 3B).

### Numerical value of the three operands in incorrectly answered problems

#### First operation

**Fig. 4:**
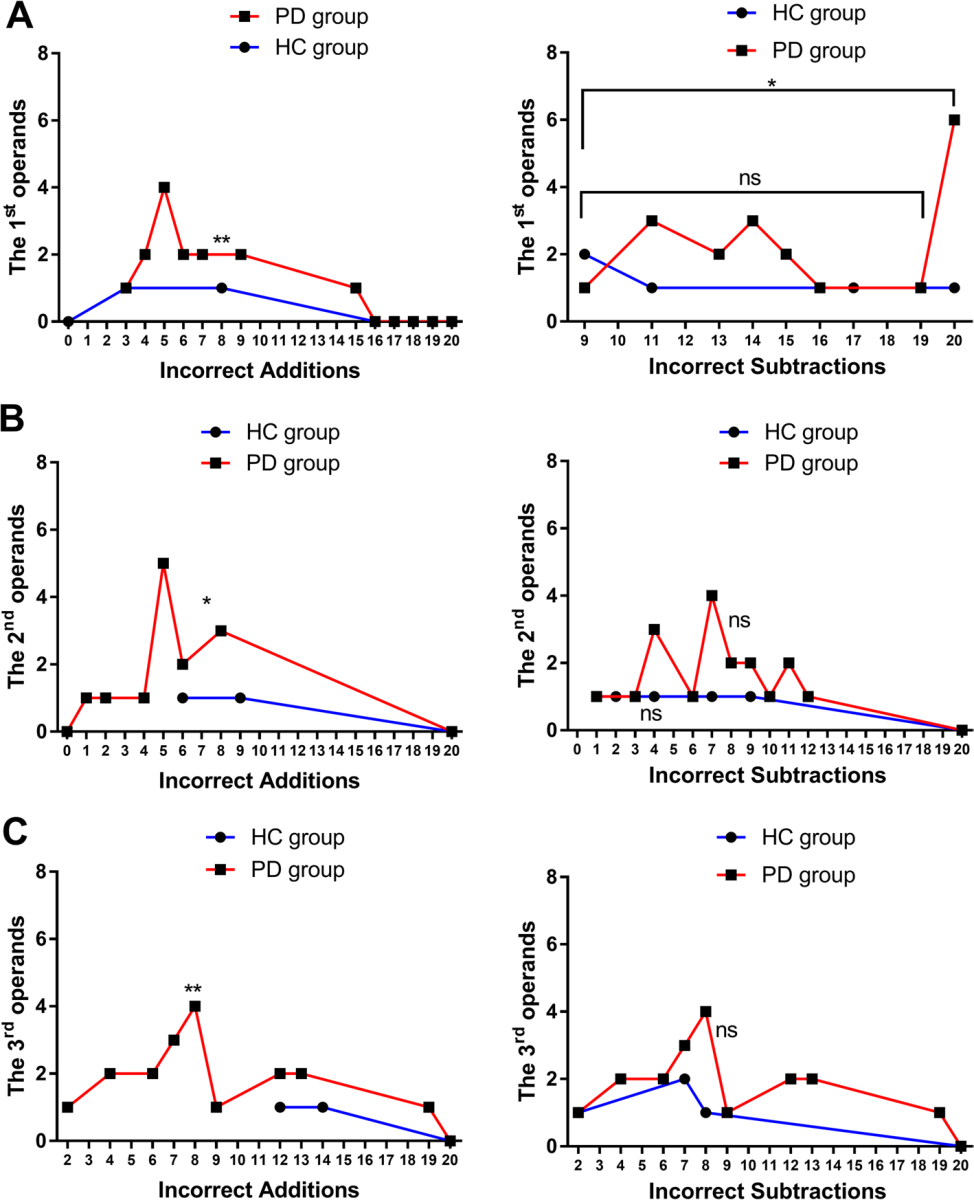
Numerical values of the three operands in the incorrectly answered problems. ns: *P* > 0.1. *: *P* < 0.1. **: *P* < 0.01

The HC group had two incorrect addition answers, while the PD group had many more incorrect addition answers, with a nonnormal distribution (*P* = 0.0088). Regarding incorrect subtraction answers, there were four in the HC group; the PD group exhibited errors in almost all values of the operand, especially for the value of 20. The values of operands in problems that were incorrectly answered were 15.53 ± 3.77 on average and were nonnormally distributed (*P* = 0.0196). When 20 was excluded as a value for the operand, the rest of the numbers (i.e., values 0–19) in problems that were incorrectly answered exhibited a normal distribution (*P* = 0.7594), with an average value of 13.46 ± 2.60 (see Fig. 4A).

#### Second operand

Regarding the incorrectly answered addition problems, the HC group made two mistakes; the PD group exhibited more errors, with a nonnormal distribution (*P* = 0.0196). Regarding the subtraction problems, the average values of the operands of the HC group and the PD group were 6.66 ± 2.51 and 7.75 ± 2.51, respectively, and both exhibited a normal distribution (*P* = 0.7804, 0.3657) (see Fig. 4B).

#### Third operand

Incorrectly answered addition problems were observed twice in the HC group. The PD group exhibited more incorrect answers, with a nonnormal distribution (*P* = 0.0087), and an average value of the operand of 10.08 ± 3.54. Regarding subtraction problems, the HC group exhibited four errors. The PD group exhibited more errors, with a normal distribution (*P* = 0.170), and an average value of the operand of 8.50 ± 4.04 (see Fig. 4C).

### Correlation between verbal repetition and incorrect answers

There was no significant correlation between verbal repetition and incorrect answers in the auditory tasks (*P* > 0.05).

## Discussion

### Cognitive overload: changes in gait parameters

In the 2-min SWT, the most significant differences between the two groups occurred in the parameters of the upper limbs and some of the lower limbs. The PD group had a slower arm swing, reduced range of arm motion, slower speed, smaller stride length, smaller foot strike angle, and less waist motion. In the DT, the group differences in the parameters of the arms and trunk did not change, except for the range of arm motion, and some of the waist parameters decreased to slightly degree (0.001 < *P* < 0.01). In other words, the movements of the arms, trunk, and waist were not greatly affected by cognitive load in patients with PD and HCs. However, the lower limb parameters were substantially affected. During the DT, the patients with PD walked more slowly and sometimes stopped unconsciously to pay more attention to mental calculation and the verbal response. Previous studies have also indicated that maintaining movement while talking is challenging for patients with PD [23, 24], and freezing is one of the indicators of cognitive overload [25].

### Cognitive overload: changes in AAS task performance

In the 2-min SAT, the PD group completed the AAS task slowly and accurately without interruption or other abnormal performance aspects indicating cognitive overload. This result indicates that for patients with PD, the cognitive load of the AAS task (with all values in the range of 0–20) did not exceed their cognitive ability. Notably, the PD group exhibited more errors in the first half of the process. As this is unlikely to be due to fatigue of calculation, it may be attributed to cognitive impairment of the patients. In the 2-min WADT, the accuracy and speed of calculation in the PD group decreased significantly, but the patients did not exhibit unconscious cessation of the AAS task (as opposed to walking). Thus, the AAS task was the priority task for patients with PD. The accuracy of calculation was the more sensitive indicator of cognitive overload for patients with PD.

### Cognitive overload: performance on the walking-AAS DT

In the DT, both the motor and cognitive performances of the two groups worsened compared to those in single tasks, and errors were observed in each part of the DT in both groups. Therefore, this paradigm (the DT) was successful, as DTs consist of three elements [12]: are carried out simultaneously, engage the same brain resources, and yield sufficient sensitive indicators. In the present study, cognitive overload was indicated by task failure or reduced performance in one of the tasks.

### Influencing factors of the cognitive load of arithmetic tasks

#### Types of mental calculation strategies

Subtraction errors were more likely than addition errors in both groups. Addition and subtraction involve different mental processes; additionally, subtraction with borrowing increases the difficulty level [26, 27]. As a result, to impose a constant cognitive load, addition and subtraction problems, especially for subtraction problems with borrowing, should not be combined in a sequential arithmetic task in the DT.

#### Numerical value of operands

Subtraction problems with a first operand equal to 20 or approximately 13, a second operand of approximately 7, or a third operand of approximately 9 were more likely to be answered incorrectly. Previous studies have shown that the calculation difficulty of addition and subtraction problems is mainly determined by the second operand in healthy people. The closer the value of the second operand is to 9, the more difficult the problem; when this value is below 4, the problem becomes easy because subjects can recall the results from memory [28, 29].

## Conclusions

In this study, a single arithmetic task, a single walking task, and a walking–arithmetic DT were performed by patients with PD and matched HCs. The DT complied with the design principles of DTs. However, even if the value of each operand was below 20, cognitive overload was observed in patients with PD. This mainly manifested in the failure of gait control, indicated by parameters of the lower limbs, and inaccurate calculation. To impose a constant cognitive load, addition and subtraction problems, especially subtraction problems with borrowing, should not be combined to create a sequential arithmetic task for DTs. Additionally, problems with the first operand equal to 20 or approximately 13, the second operand approximately 7, or the third operand approximately 9 should be excluded from the AAS task. A larger sample size is needed to identify the relative difficulty level of addition problems among patients with PD because the difficulty may also be impacted by the operational sequence and numerical magnitude [30].

### Limitations

The sample sizes of the HC group and PD group were insufficient to obtain enough incorrect answers to addition problems for analysis. The AAS task was the primary focus of attention for patients with PD, prioritized above maintenance of walking.

## Data Availability

The datasets generated during the current study are available in Mendeley Data. The data identification number is: https://doi.org/10.17632/vfvfprdyxp.1. The direct URL to data is: https://data.mendeley.com/datasets/vfvfprdyxp/1.

## Abbreviations

PD: Parkinson’s disease
PD group: Parkinson’s disease group
HC group: Elderly healthy control group
DT: Dual task
2-min SAT: 2-Min single arithmetic task
2-min SWT: 2-Min single walking task
2-min WADT: 2-Min walking–arithmetic dual task
AAS: Auditory addition and subtraction
